# Dexmedetomidine in Bariatric Surgery: A Systematic Review and Meta-Analysis of Its Effects on Postoperative Pain and Postoperative Nausea and Vomiting

**DOI:** 10.3390/jcm14030679

**Published:** 2025-01-21

**Authors:** Reem Altamimi, Danah Alnajjar, Rawan Bin Salamah, Joana Mandoorah, Abdulaziz Alghamdi, Reema E. Aloteibi, Lamya Almusharaf, Bader Albabtain

**Affiliations:** 1 College of Medicine, Princess Nourah bint Abdulrahman University, Riyadh 14256, Saudi Arabia; rawanbinsalamah@gmail.com (R.B.S.); laalmusharaf@pnu.edu.sa (L.A.); 2 College of Medicine, Taibah University, Medina 42361, Saudi Arabia; alnajjardanah@gmail.com; 3 Department of Health Sciences, King Saud bin Abdulaziz University, Riyadh 11481, Saudi Arabia; joanamandoorah@gmail.com (J.M.); dammasa99@gmail.com (A.A.);; 4 Ministry of National Guard Health Affairs, Riyadh 11426, Saudi Arabia; albabteanba@mngha.med.sa

**Keywords:** dexmedetomidine, anesthesia, intraoperative analgesia, postoperative pain management, postoperative nausea and vomiting (PONV), perioperative care

## Abstract

**Background:** Bariatric surgery is associated with significant postoperative challenges, including pain and nausea. Dexmedetomidine (Dex), an alpha-2 adrenergic agonist, is commonly used to manage pain and postoperative nausea and vomiting (PONV) in various surgical settings. This meta-analysis evaluates the efficacy of Dex in bariatric surgery patients, focusing on postoperative pain intensity, opioid consumption, and PONV. **Methods:** We conducted a systematic review and meta-analysis of randomized controlled trials (RCTs) published between 2010 and 2023, assessing Dex use during or after bariatric surgery. Studies comparing Dex to placebo or standard care were included. Data extraction was performed independently by two reviewers, and statistical analysis was conducted using a random-effects model. Study quality was assessed using the Cochrane Risk of Bias tool. **Results:** Six RCTs (485 participants) met the inclusion criteria. Dex significantly reduced intraoperative fentanyl use (SMD −1.33, 95% CI [−2.19, −0.47], *p* = 0.002). Pain scores showed mixed results, with some studies reporting lower pain intensity in the Dex group, while others found no significant difference compared to morphine or placebo. PONV scores were generally lower in the Dex group (*p* = 0.01) compared to placebo and morphine. No significant differences were found in morphine consumption (SMD −1.13, 95% CI [−2.24, 0.01], *p* = 0.05) or recovery time. **Conclusions:** Dexmedetomidine appears to reduce opioid requirements and postoperative nausea in bariatric surgery patients. However, the variability in pain management outcomes suggests that further well-designed RCTs are needed to confirm its overall efficacy. The findings are based on moderate-quality evidence, and further research should aim to standardize dosing protocols and patient populations.

## 1. Introduction

Bariatric surgery has emerged as one of the most rapidly expanding surgical procedures, with approximately 720,000 interventions carried out in 2018 [1]. As the incidence of morbid obesity escalates, the frequency of bariatric surgeries also rises, encompassing procedures such as sleeve gastrectomy (SG) and Roux-en-Y gastric bypass (RYGB). These surgical interventions provide numerous advantages, including life expectancy enhancements, weight loss promotion, and a reduction in various metabolic comorbidities [2,3]. However, achieving optimal outcomes depends on thorough preoperative assessment and optimization of these patients, as emphasized in recent European guidelines for elective noncardiac surgery. This process is essential for lowering perioperative risks and improving postoperative patients’ recovery [4].

Nevertheless, in spite of these benefits, up to 61.4% of patients report experiencing persistent postoperative pain following bariatric surgeries, particularly abdominal discomfort, as indicated by several studies [5,6,7,8,9,10,11,12].

According to The International Association for the Study of Pain (IASP), persistent postoperative pain is defined as clinical discomfort that persists for more than two months following surgery, with the exclusion of previous pain disorders or recurrent infections. This type of pain is classified as a continuation of acute postoperative pain that can appear after an asymptomatic period, and it has distinct characteristics or greater severity than preoperative pain, according to the International Classification of Diseases (ICD). In recognition of the differences in recovery timeframes between surgeries, the ICD establishes a three-month postoperative period for persistent postoperative discomfort. The location of this pain is usually abdominal in bariatric surgeries [13,14,15].

The effective management of acute postoperative pain is of paramount importance, as it may contribute to a reduction in the risk of developing chronic pain [16].

Furthermore, bariatric surgery, especially procedures that involve the stomach or intestines, is associated with a substantially higher incidence of postoperative nausea and vomiting (PONV) [17]. As the population of morbidly obese patients undergoing bariatric surgery continues to increase, the management of these postoperative complications becomes progressively critical.

Dexmedetomidine (^®^), a highly selective alpha-2 adrenergic agonist, has garnered extensive utilization due to its sedative, anxiolytic, sympatholytic, and analgesia-sparing properties. Furthermore, it has been demonstrated to diminish the necessity for anesthetics and opioids during the perioperative period [18].

In light of these attributes, dexmedetomidine may represent a promising approach for managing both postoperative pain and postoperative nausea and vomiting (PONV) in bariatric surgery surgeries.

This systematic review aims to fill this gap by evaluating the efficacy of dexmedetomidine in mitigating postoperative pain and preventing PONV specifically in bariatric surgery patients. By analyzing randomized controlled trials (RCTs) conducted between January 2010 and October 2023, this review seeks to provide evidence-based insights into its role in improving postoperative outcomes in this growing patient population.

## 2. Materials and Methods

This research was executed in accordance with the Preferred Reporting Items for Systematic Reviews and Meta-Analyses (PRISMA) guidelines [19]. It is registered in the International Prospective Register of Systematic Reviews (PROSPERO) (ID: CRD42023484709) because this study was a systematic review; formal ethical approval was not required.

### 2.1. Eligibility Criteria

This review encompasses randomized controlled trials that meet the following criteria: (1) adults aged 18 or older undergoing any form of bariatric surgery, including but not restricted to gastric bypass, sleeve gastrectomy, adjustable gastric banding, and biliopancreatic diversion; (2) trials that examined dexmedetomidine used as an intervention during or after bariatric surgery; (3) trials that compared dexmedetomidine with standard perioperative care. Case reports, series, and studies published in languages other than English were excluded.

### 2.2. Search and Identification of Studies

Systematic searches were conducted for articles in journals listed on PubMed, Web of Science, Google Scholar (PERSONALIZATION), and ProQuest, from inception to October 2023, using the following terms: (“Dexmedetomidine” OR “Precedex”) AND (“Bariatric Surgery” OR “Gastric Bypass” OR “Obesity Surgery” OR “Weight Loss Surgery”) AND (“Postoperative Pain” OR “Analgesia” OR “Pain Management” OR “Pain Control”) AND (“Postoperative Nausea and Vomiting” OR “PONV” OR “Nausea and Vomiting, Postoperative” OR “Postoperative Nausea” OR “Postoperative Vomiting”). Retrieved citations were imported into an Excel Sheet.

### 2.3. Screening and Study Selection

**Figure 1:**
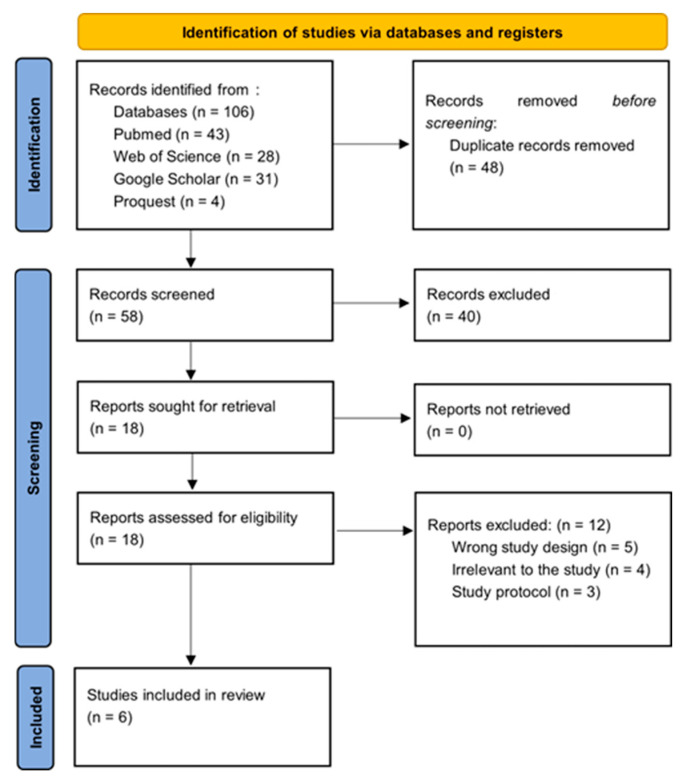
PRISMA flow diagram.

After the initial search, the studies identified were systematically uploaded to the Rayyan platform for the purposes of de-duplication and preliminary screening predicated upon their titles and abstracts. This process was conducted independently by two authors, with any discrepancies being resolved by a third party. The articles that were deemed appropriate were subsequently retrieved and subjected to thorough full-text screening by three independent reviewers, who assessed the articles in accordance with predetermined inclusion and exclusion criteria. Discussions were held to address any disputes regarding eligibility, and when required, a third researcher was consulted to achieve a consensus. Full-text articles that met the eligibility criteria were procured and independently evaluated for inclusion and exclusion by two pairs of reviewers. For an article to be considered for inclusion, it necessitated approval from the independent reviewers. Refer to Figure 1.

### 2.4. Data Extraction

Four authors independently extracted the data pertaining to this study. The acquired information included study characteristics, participant details, specifics of the interventions, data regarding comparison/control groups, measured outcomes, significant findings, reported adverse events, and identified limitations of the study. These data were systematically organized within a preformatted Excel workbook developed using Microsoft Excel 2021.

### 2.5. Statistical Analysis

Data entry and analysis were conducted using Review Manager, version 5.4. (The Cochrane Collaboration, Oxford, UK). If not provided, a mean’s standard deviation (SD) was estimated from CI limits or standard mean difference. The data were treated as means for trials reporting medians, and the SD was obtained by dividing the interquartile range by 1.35, as suggested in the Cochrane Handbook for Systematic Reviews of Interventions [20]. The effect size of the continuous outcomes was reported as standard mean difference (SMD), and the precision of effect size was reported as a 95% confidence interval (CI). The DerSimonian and Laird random-effects model was used to compute SMD [21]. We used the Cochrane Q tests and I_2_ statistics to evaluate the heterogeneity and inconsistency of treatment effects across trials, respectively. Statistical significance was set at *p*-value < 0.01 for Cochrane Q tests. The I_2_ statistics represent the degree of heterogeneity and the proportion of treatment variation independent of sampling error. Moderate heterogeneity assumed as I_2_ was 30% to 60%. Neither the Funnel plot nor Egger’s test for publication bias detection was performed because the number of the studies included is less than 9.

## 3. Results

This systematic review aimed to comprehensively assess the effects of dexmedetomidine (Dex) on postoperative pain intensity, anesthetic requirements, and incidence/severity of postoperative nausea and vomiting (PONV) during bariatric surgery. The review’s secondary outcomes included the recovery profile, hemodynamic stability, and adverse effects. The review included six randomized controlled trials (RCTs) in this PRISMA flowchart (Moher et al. 2009) [19] published from 2007 to 2023 [18,22,23,24,25,26,27,28,29,30,31].

The included studies consisted of patients who underwent various bariatric surgeries, with a sample size of 56 and 119, for a total of 485. This diversity in surgical procedures, including gastric banding, gastric bypass, sleeve gastrectomy, Roux-en-Y gastric bypass, LRYGB, and conversions (removal of GB then LRYGB), ensures that the findings apply to a wide range of cases.

Table 1 highlights the key features of the studies included.

### 3.1. Data Analysis

#### 3.1.1. Postoperative Pain Intensity

Five of our included studies reported postoperative pain intensity scores. Khalil BN et al. [22] found that ketamine resulted in a decrease in postoperative pain intensity compared to Dex 0.5 mcg, ketamine, and control groups. The decrease was statistically significant at 0 min (*p* < 0.001), 30 min (*p* < 0.001), 60 min (*p* = 0.017), and 12 h (*p* = 0.012) of infusion in the Ketamine group, but not in the Dex group. Similarly, Zeeni C et al. [23] found that the pain scores were similar in the Dex and morphine groups. Dex VAS scores were compared to morphine in the Abu-Halaweh S et al. [24] study, where group Dex 0.3 mcg (63%) had a higher severe VAS score (>70) than the morphine group (20%) (*p* = 0.0007). However, the drug-by-time interaction was not significant for (VAS) (*p* = 0.45). Ziemann-Gimmel P et al. [25], compared TIVIA (0.1–0.3 mcg Dex) and classic groups, which revealed no significant difference in the acceptable NPS pain scores (*p* = 0.53) and NPS pain scores on arrival at the ward (*p* = 0.66).

Tufanogullari B et al. [18] used a different dosing regimen of Dex (0.2, 0.4, and 0.8 mcg) compared to placebo. The findings showed that VAS pain scores in the post-anesthesia care unit (PACU) and the average on POD 1, 2, and 7 did not differ significantly among the three Dex groups and the control group. However, Hassan S B et al. [26], who used 0.4 mcg Dex, concluded that pain scores at one hour and two hours were significantly lower in the Dex group compared to placebo.

#### 3.1.2. Postoperative Nausea and Vomiting (PONV)

Three of the five studies assessed PONV with Dex found less significant PONV episodes than placebo/control. Abu-Halaweh S et al. [24] reported that significantly more patients had nausea or vomiting in the morphine. However, there was no difference between groups for vomiting (10.0% Group Dex vs. 23.3% Group morphine, *p* = 0.1659). Furthermore, Ziemann-Gimmel P et al. [25] assessed the severity of nausea, demonstrating that opioid-free total intravenous anesthesia (TIVA) was associated with a large decline in the relative risk of PONV compared with the balanced anesthesia group. The study findings revealed that the severity of nausea differed in both groups (*p* < 0.02). In the Classic group, more patients experienced retching than in the TIVA group (Dex group). All patients who reported experiencing retching characterized their level of nausea as severe. Among the seven patients in the Classic group who reported retching, five individuals indicated that they experienced vomiting.

Tufanogullari B et al. [18] found that the overall incidences of postoperative emetic symptoms during the first 24 h after surgery were reduced in the Dex (0.2, 0.4, and 0.8) groups compared with the control group. Similarly, the need for rescue antiemetic drugs was significantly reduced in all three Dex groups. Hassan S B et al. [26] found that the incidence and severity of PONV showed no statistically significant difference between the groups.

On the other hand, Khalil BN et al. [22] found that PONV scores were lower, but non-significant, in the dexmedetomidine group (8/30) than in the ketamine (13/30) and control group (16/30). Similarly, Zeeni C et al. [23] found that regarding NRS nausea scores, episodes of emesis were found to be 0 (IQR 0 to 0) for the Dex group vs. 0 (IQR 0 to 1) for the morphine group, and rescue antiemetic use was 0 (IQR 0 to 0) for the Dex group vs. 0 (IQR 0 to 1) for the morphine group. Although the IQR was higher in the morphine group, the difference did not reach statistical significance.

#### 3.1.3. Analgesic Requirements

Different regiments were integrated into the studies where opioids such as morphine or fentanyl, acetaminophen, or non-steroidal anti-inflammatory drugs were administrated. For instance, Khalil BN et al. [22] demonstrated significantly less cumulative morphine consumption in the ketamine group (3 ± 3 mg) compared with the other Dex (5 ± 4 mg) and control groups (7 ± 3 mg) (*p* < 0.001). In addition, Zeeni C et al. [23] found no significant differences in morphine consumption in the PACU or at 24 h after surgery. Abu-Halaweh et al. [24] also revealed no differences in mean rescue paracetamol and morphine requirements, mean supplemental morphine requirements, or the number of patients who required paracetamol for moderate pain. The total amount of morphine administered was lower in the Dex group (6.1 ± 3.1 mg) compared to the morphine group 72.9 ± 2.2 mg (*p* < 0.0001).

Ziemann-Gimmel P et al. [25] also found a non-significance in average morphine doses postoperatively, contrary to Tufanogullari B et al. [18] or Hassan S B et al. [26] studies.

#### 3.1.4. Total Amount of Intraoperative Fentanyl (Microgram)

**Figure 2:**
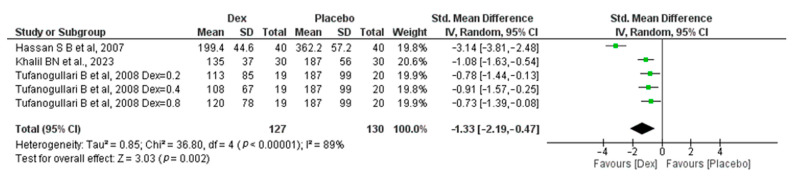
Forest plot of comparison: Dex vs. Placebo, outcome: Total amount of Intraoperative Fentanyl [18,22,26].

The meta-analysis includes three studies [18,22,26] (a study conducted by Tafanogullari B et al.,2008 with three different doses). A total of 127 patients in the Dex group and 130 in the placebo group revealed a significant difference between the two groups, with an SMD of −1.33 (95% CI, −2.19, −0.47; *p*-value = 0.002) in favor of the placebo group. Significant heterogeneity was found (I_2_ = 89%, *p*-value < 0.00001) Figure 2.

#### 3.1.5. Total Amount of Morphine Consumption (Microgram)

**Figure 3:**
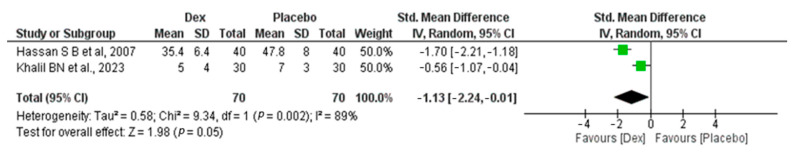
Forest plot of comparison: Dex vs. placebo, outcome: Total amount of morphine consumption [22,26].

Regarding the total amount of morphine consumption, the meta-analysis includes two studies, with 70 patients in the Dex group and 70 in the placebo group, which revealed a non-significant difference between the two groups, with SMD −1.13 (95% CI (−2.24, 0.01), *p*-value = 0.05). Significant heterogeneity was found (I^2^ = 89%, *p*-value = 0.002) Figure 3.

#### 3.1.6. Length of Surgery (Min)

**Figure 4:**
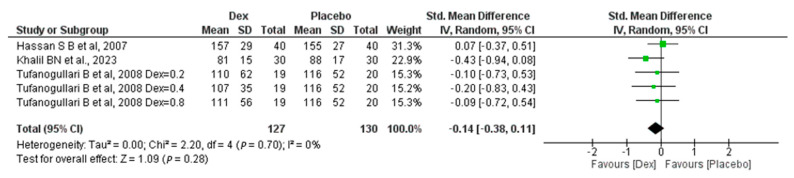
Forest plot of comparison: Dex vs. Placebo, outcome: Length of surgery [18,22,26].

Our meta-analysis includes three studies, one of which was conducted by Tafanogullari B et al. in 2008 [18] with three different dosages. In this study, 127 patients were in the Dex group and 130 in the placebo group, revealing a non-significant difference between the two groups, with (SMD −0.14, 95% CI, −0.38, 0.11, *p*-value = 0.28). A non-significant low heterogeneity was found (I^2^ = 0%, *p*-value = 0.7) Figure 4.

#### 3.1.7. Time to Extubation (Min)

**Figure 5:**
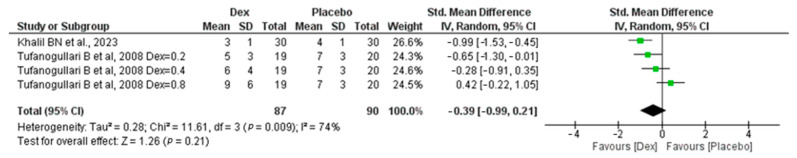
Forest plot of comparison: Dex vs. Placebo, outcome: Time to extubation [18,22].

Regarding time to extubation, among three studies, 87 patients in the Dex group and 90 in the placebo group revealed a non-significant difference between the two groups, with (SMD −0.39, 95% CI, −0.99, 0.21, *p*-value = 0.21). However, significant heterogeneity was found (I^2^ = 74%, *p*-value = 0.009) Figure 5.

#### 3.1.8. Alertness/Sedation MOASS

Zeen C et al. revealed that MOASS scores significantly differed between the two groups. Khalil et al. [22] found that Dex marginally improved MOASS scores. Our meta-analysis includes these two studies, in which 114 patients were in the Dex group and 118 were in the ketamine group, revealing a non-significant overall difference between the two groups, with (SMD 0.16, 95% CI, −0.57, 0.89, *p*-value = 0.66). A significant high heterogeneity was found (I^2^ = 83%, *p*-value = 0.003).

#### 3.1.9. Hospital Length of Stay

**Figure 6:**
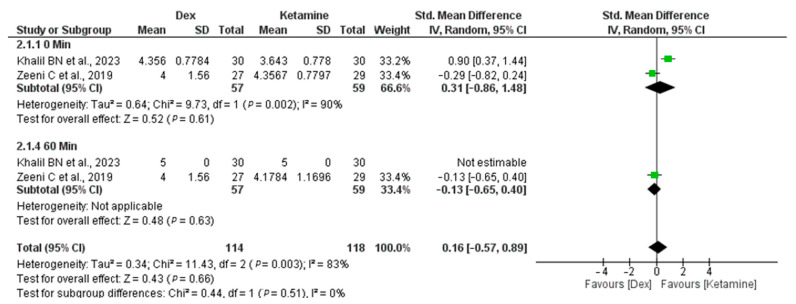
Recovery time and length of hospital stay did not differ significantly among the Dex and control groups in the studies [22,23].

Regarding hospital length of stay, two studies (Khalil et al. and Zeeni et al.) compared Dex and Ketamine [22,23]. The analysis shows no clear difference between Dexmedetomidine and Ketamine for the measured outcome at 10 or 60 minutes. High heterogeneity (especially at 10 minutes) indicates variability in study results, requiring further investigation or consideration of study design differences (I^2^ = 83%, *p*-value = 0.003) Figure 6.

### 3.2. Subgroup Analysis

In order to explore the differential effects of dexmedetomidine on postoperative pain and PONV, a subgroup analysis was conducted and stratified by several procedure types (ex. sleeve gastrectomy, Roux-en-Y gastric bypass, and gastric band).

#### 3.2.1. Postoperative Pain

1. Sleeve Gastrectomy

Research shows that dexmedetomidine considerably lessens the severity of pain in patients having sleeve gastrectomy. For instance, Hassan et al. [26] demonstrated that dexmedetomidine infusion decreased the need for postoperative opioids, which was associated with better pain scores (VAS reduced by about 30%) in patients who had laparoscopic sleeve gastrectomy. Furthermore, Tian et al. [32] validated similar results, emphasizing quicker recuperation and a reduced need for analgesics.

Finally, according to Zeeni et al. [23], administering dexmedetomidine before the end of a laparoscopic sleeve gastrectomy offers comparable postoperative analgesia to morphine while maintaining a superior hemodynamic profile.

2. Gastric Bypass

Dexmedetomidine showed a pronounced effect in reducing postoperative pain in gastric bypass patients. Singh et al. [33] reported a 35% reduction in opioid consumption and better pain control compared to standard regimens. Another meta-analysis (e.g., Zhang et al. [29]) also highlighted that this demographic benefited from long-lasting analgesic benefits, most likely due to the sympatholytic qualities of dexmedetomidine.

3. Gastric Band

Although limited data is available for this subgroup, studies such as Kruthiventi et al. suggest that dexmedetomidine has a moderately effective pain management impact. This may be due to the less invasive nature of gastric band procedures compared to sleeve gastrectomy and gastric bypass.

#### 3.2.2. Postoperative Nausea and Vomiting (PONV)

1. Sleeve Gastrectomy

Mostafa et al. [31] demonstrated a 25% drop in PONV incidence with dexmedetomidine compared to controls, indicating a significant reduction in PONV. The mechanism is likely linked to reduced catecholamine release and enhanced parasympathetic tone.

2. Gastric Bypass

While Chang et al. [34] reported some benefits, the overall reduction in PONV was less significant, possibly due to the shorter procedure duration and minimal surgical trauma.

3. Gastric Band

Although limited data is available for this subgroup, research such as Kruthiventi et al. [30] suggests that dexmedetomidine has a moderately effective pain management impact. This may be due to the less invasive nature of gastric band procedures compared to sleeve gastrectomy and gastric bypass.

The subgroup analysis underscores the consistent benefits of dexmedetomidine across all procedure types. However, the magnitude of its effects appears greatest in gastric bypass patients, possibly because of their higher baseline risks and increased postoperative stress.

#### 3.2.3. Analgesic Requirements

In all subtypes of surgery, there was less morphine consumption in the ketamine group reported by Khalil BN et al. [22] in contrast to the Dex and control groups.

1. Sleeve Gastrectomy

Only less amount of morphine consumption in the ketamine group was reported.

2. Gastric Bypass

According to Ziemann-Gimmel P et al. [25], average hydromorphone doses were equal in the postoperative period. On the other hand, Hassan S B et al. [26] reported less amount of PCA morphine given at 2 h in the PACU and POD in the Dex group.

3. Gastric Band

Tufanogullar B et al. [18] reported less amounts of rescue fentanyl given in the PACU in the Dex group.

#### 3.2.4. Total Amount of Intraoperative Fentanyl (Microgram)

**Figure 7:**
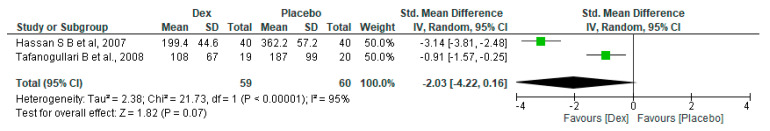
Forest plot of comparison: Dex vs. Placebo, outcome: Total amount of Intraoperative Fentanyl (Dose = 0.4 mcg/kg/h) [18,26].

The meta-analysis includes two studies [18,26] involving 40 patients in the Dex (Dose = 0.4 mcg/kg/h) group and 20 in the placebo group, revealing a non-significant difference between the two groups, with an SMD of −2.03 (95% CI, −4.22, −0.16, *p*-value = 0.07). Significant heterogeneity was found (I^2^ 95%, *p*-value < 0.00001) (Figure 7).

#### 3.2.5. Length of Surgery (Min)

**Figure 8:**
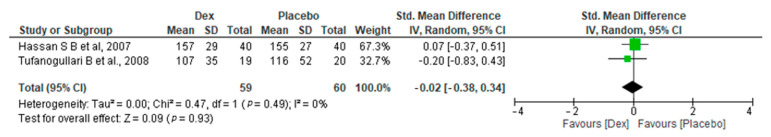
Forest plot of comparison: Dex vs. Placebo, outcome: Length of surgery. (Dose = 0.4 mcg/kg/h) [18,26].

Our meta-analysis includes two studies [18,26] on 40 patients in the Dex (Dose = 0.4 mcg/kg/h) group and 20 in the placebo group, revealed a non-significant difference between the two groups, with (SMD −0.20, 95% CI, −0.38, 0.34, *p*-value = 0.93). A non-significant low heterogeneity was found (I^2^ = 0%, *p*-value = 0.49) (Figure 8).

### 3.3. Risk of Bias Assessment

**Figure 9:**
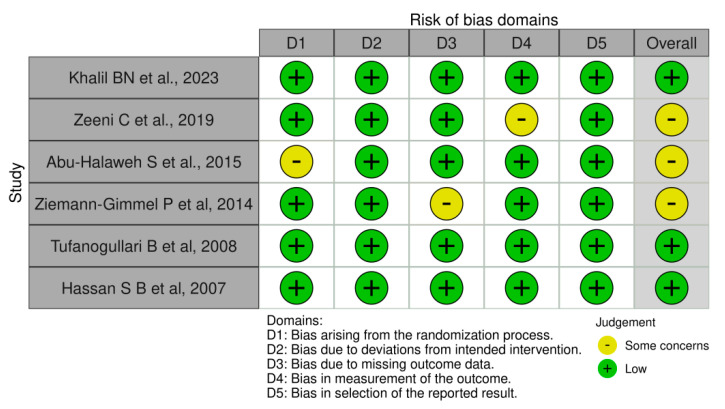
Risk of bias assessment (ROB2) of included studies: Khalil BN et al. 2023, Zeeni C et al. 2019, Abu-Halaweh S et al. 2015, Ziemann-Gimmel P et al. 2014, Tufanogullari B et al. 2009, and Hassan S B et al. 2007 [18,22,23,24,25,26].

Risk of bias traffic light plot assessment of the included studies (Figure 9).

## 4. Discussion

Elective surgery for morbid obesity patients most commonly involves weight loss (bariatric surgery). Such intricate surgical operations may be associated with postoperative pain [35]. Anesthetic management of these patients must be taken into careful consideration. An α2-adrenergic receptor agonist, dexmedetomidine, is recently often being used preoperatively due to its favorable characteristics. These traits include anxiolysis, sedation, and minimal effect on breathing [32]. In fact, clinical studies have also demonstrated its effectiveness in blood pressure control and the suppression of salivary gland secretions [34].

This systematic review and meta-analysis of six RCTs and 485 participants demonstrated that Dex administration during bariatric surgery revealed a significantly lower dose of intraoperative fentanyl in patients prescribed Dex than in control groups with patients prescribed placebo. A previous meta-analysis conducted in 2022 showed that Dex infusion in opioid-sparing analgesia had a major role in postoperative analgesia after bariatric surgery; similarly to our findings, the heterogeneity was high [18]. Other non-significant findings were seen in total morphine consumption, length of surgery, time to extubation, MOASS, recovery time, and length of stay.

The primary and secondary outcomes assessed in this review were inconsistent in terms of significance and magnitude. The reason for this inconsistency is the differences in the sample characteristics and dosing regimens for the intervention and control groups. These differences are crucial in understanding the current knowledge of bariatric surgery and analgesia.

Regarding primary outcomes, postoperative pain intensity ranged between the included studies, as two showed non-significant differences between Dex and the comparable group [6,18,25], a similar effect of Dex compared to morphine [23], one favoring ketamine over Dex in reducing pain intensity [22], and one indicated significantly higher efficacy of Dex over placebo at two time points [13]. However, one study opposed these results, reporting higher pain severity in the Dex group compared to morphine [24]. In 2017, another meta-analysis was conducted to evaluate the safety profile of Dex; morbidly obese patients receiving perioperative Dex infusions had overall better pain control in comparison to conventional analgesic regimens [33].

Regarding analgesic requirements, Dex reduced opioid requirements in some studies [18,22,24,26]. Studies used opioids such as morphine and fentanyl, acetaminophen, or NSAIDs for pain management. A retrospective study conducted in 2007 discovered that Dex infusion perioperatively was found to be safe and could help to reduce narcotic requirements and limit the post-operational length of stay following a laparoscopic bariatric procedure [26]. These findings align with our review and could suggest that Dex has a postoperative benefit among bariatric surgery patients regarding pain score and analgesic demands.

Furthermore, PONV scores were assessed among the included studies, revealing lower scores compared to the ketamine and control groups as assessed by Khalil et al. [22]. Similarly, Abu-Halaweh et al. [24] found that the incidence of nausea and vomiting decreased among the Dex group in comparison to morphine [24]. This was consistent with the study by Tufanogullari et al. [18]. However, this was inconsistent with Hassan et al. [26] and Zeeni et al. [23], as they revealed no significant difference between the Dex group, morphine, and placebo. In contrast, a propensity-weighted analysis conducted in 2020 showed that Dex-based anesthesia was associated with reduced opioid and volatile agent use but not associated with a reduction in PONV [22].

Moreover, Khalil BN et al. [22] and Hassan SB et al. [26] assessed the recovery profile, revealing improved sedation scores and a better postoperative recovery profile. Similarly, in a prospective RCT conducted in 2021, patients undergoing laparoscopic bariatric surgeries who were administered Dex experienced safe and effective outcomes regarding their recovery profile [34].

In addition, the included studies addressed some cardiac events. For instance, Khalil BN et al. [22] showed no significant difference in the MAP between the Dex and the control groups. HRs were significantly lower in the Dex group than in the other groups [22]. In addition, Zeeni C et al. [23] found that SBP and DBP were significantly lower in the Dex group than in the morphine group. HR was also significantly lower in the Dex group compared to the morphine group at extubation [23]. Hassan et al. showed that the MAP and HR significantly decreased in the Dex group compared with the placebo group [26]. Similarly, another study confirmed this finding, revealing a significant reduction in HR and MAP in the Dex group in non-diabetic morbidly obese patients undergoing laparoscopic bariatric surgery [23]. Tufanogullari B et al. revealed that MAP values in the PACU during the first 45 min were significantly lower in the Dex three groups [18]. In contrast, Ziemann-Gimmel P et al. found two adverse events in the TIVA group, including a second-degree AV block and hypotension in the PACU [25]. Abu Halawa et al. found a significant time-by-group interaction for HR over 24 h hours (*p* = 0.0038) [24].

Upon examining bias domains, three studies showed no concerns, indicating adherence to high standards in randomization, blinding, and outcome assessment. In contrast, four studies raised some issues regarding unclear randomization, missing outcome data, and outcome measurement, which require caution in interpreting results as these factors may affect the perceived effectiveness of the intervention.

The diverse presence of concerns leads to a moderate risk of bias (50%) as indicated by the Cochrane Risk of Bias tool version 2. This suggests that although the findings hold value, they should be approached with skepticism regarding the evaluation of the intervention’s true efficacy. Future research ought to address the identified concerns to improve the reliability of the results.

## 5. Limitations

This systematic review and meta-analysis examining the effects of dexmedetomidine (Dex) on postoperative outcomes in bariatric surgery patients has several limitations that must be considered when interpreting the results. First, the included studies exhibited significant heterogeneity, particularly in terms of sample sizes, dosing regimens, and the types of bariatric surgeries performed. This variability makes it difficult to draw definitive conclusions across all outcomes, and despite efforts to conduct sensitivity analyses, the high heterogeneity, especially regarding opioid consumption and postoperative pain scores, limits the generalizability of the findings.

Additionally, the small number of studies (n = 6) included in this meta-analysis precluded the use of trial sequential analysis (TSA), which could have assessed the robustness and statistical power of the results. The lack of TSA is a notable limitation, as such an analysis might have provided more precise estimates and helped confirm the reliability of our findings. Furthermore, publication bias was not formally assessed due to the limited number of studies available, which hampers the ability to apply standard methods like funnel plots or Egger’s test. The small sample size restricts the power of these assessments and raises the possibility of publication bias, which may affect the overall reliability of the conclusions.

Finally, while we adhered to PRISMA guidelines to the best of our ability, variability in study quality and reporting may have introduced bias in some of the included trials. Future research with larger sample sizes, more standardized dosing regimens, and improved study designs is necessary to validate these findings and provide a clearer understanding of the role of dexmedetomidine in the management of postoperative outcomes in bariatric surgery patients.

## 6. Conclusions

This systematic review and meta-analysis studied the efficacy of Dex among bariatric surgery patients in attenuating several postoperative outcomes. The results of this meta-analysis revealed significantly less need for intraoperative fentanyl with the use of Dex intraoperatively. However, some outcomes showed non-significant differences. Despite that, Dex has proven its efficacy in reducing PONV and pain intensity. Due to the obvious heterogeneity seen, more research, especially RCTs, should be conducted to validate these findings.

## Figures and Tables

**Table 1:** Characteristics of the included studies.

Study Author	Study Aim	Study Design	Randomization Technique (Intervention Details)	Population Characteristics	Type of Bariatric Surgery	Primary Outcome	Secondary Outcomes	Notes
Khalil BN et al., 2023, Egypt [22]	To study whether the choice of ketamine or dexmedetomidine (Dex) infusion would affect postoperative total morphine consumption.	Prospective, randomized, double-blinded, three-armed, controlled trial	Ketamine group: a bolus dose (0.3 mg/kg) of ketamine, followed by an infusion of (0.3 mg/kg/h). Dex group: a bolus dose (0.5 mcg/kg) of dexmedetomidine followed by an infusion of (0.5 mg/kg/h). Control group: a saline infusion. All infusions were given until 10 min before the end of surgeries.	Sample size (N) = 90 Dex: Age: Mean ±SD: 34 ± 7 BMI (kg/m^2^): 43 ± 4 Ketamine: Age: 37 ± 4 BMI: 41 ± 2 Control: Age: 34 ± 7 BMI: 43 ± 4 Comorbid: ASA II/III HM, DM, hypothyroidism	Elective laparoscopic sleeve gastrectomy	Analgesic requirements (total morphine dose) significantly less cumulative morphine consumption in the ketamine group (3 ± 3 mg) compared with the other Dex (5 ± 4 mg) and control (7 ± 3 mg) groups (*p* < 0.001).	Intraoperative fentanyl requirement: Dex decreased the need for fentanyl intraoperatively (135 ± 37 µg) compared with ketamine (160 ± 42 µg), control groups (187 ± 56 µg), *p*-value < 0.001. Modified observer’s agitation/sedation scale (MOASS) score: MOASS scores were better in the dexmedetomidine group than in the other groups, especially in the first 10 min and returned to baseline at 30 and 60 min in all groups. Postoperative pain intensity: At 0, 30, and 60 min, the ketamine group’s numerical rating scale (NRS) scores were significantly lower than those of the other groups. Time to extubation: significantly shorter in the Dex group (3 ± 1 min) than in ketamine (4 ± 1) and control (4 ± 1) groups, *p*-value = 0.005. Postoperative nausea and vomiting (PONV) scores: PONV scores were not significantly lower in the Dex group than in the other groups (*p* = 0.106).	Hemodynamic stability = Mean arterial pressure (MAP) and heart rate (HR) did not decrease significantly across the groups. The length of surgery was not significantly higher in the control (88 ± 17) group than Dex (81 ± 15) and ketamine (87 ± 15), *p* = 0.128
Zeeni C et al., 2019, Lebanon [23]	To compare the effect of Dex bolus and infusion versus morphine bolus given before the end of laparoscopic bariatric surgery.	Prospective, randomized, double-blind trial.	Group M: Morphine sulfate (bolus 0.08 mg/kg followed by a saline infusion) (n = 30) Group D: Dex (loading dose of 1 mcg kg followed by 0.5 mcg/kg/h) (n = 30). Medications were given 30 min before the end of surgery.	Sample size (N) = 56 Age (Mean ± SD) Dex: 38.04 ± 12.43 Morphine: 38.03 ± 10.44 BMI Dex: 42.14 ± 6.53 Morphine: 41.78 ± 5.86 Comorbid condition = ASA II (Hypertension and diabetes)	laparoscopic sleeve bariatric surgery	Total morphine consumption in the post-anesthesia care unit (PACU): No significant differences were found in morphine consumption in the PACU (group D 12.2 ± 5.44 mg, group M 13.28 ± 6.64 mg, *p* = 0.54).	Intraoperative morphine consumption: Group M had significantly higher morphine consumption (intraoperative plus PACU) than group D (23.48 ± 6.22 mg vs. 12.22 ± 5.54 mg, respectively, *p* < 0.01). Morphine consumption at 24 h No significant differences were found between group D 40.67 ± 24.78 mg, and group M 43.28 ± 27.79 mg, *p* = 0.75). Postoperative pain intensity = NRS pain scores were similar between groups.Incidence and severity of PONV = NRS nausea scores, episodes of emesis 0 (IQR 0 to 0) for group D vs. 0 (IQR 0 to 1) for group M, and rescue antiemetic use 0 (IQR 0 to 0) for group D vs. 0 (IQR 0 to 1) for group M were not significantly higher in group M than in D. Time to discharge readiness from the PACU was not statistically different between the groups: 78.37 ± 27.10 min for group D vs. 76.62 ± 19.92 min for group M, *p* = 0.77. Patient satisfaction = Quality of recovery scores and NRS scores for satisfaction one month post-discharge were comparable between the groups. The Median for group D was 10.00 [9.00 to 10.00] vs. 9.00 [8.00 to 10.00] for group M.	Time to first morphine requirement was not significantly different between groups (group D 8.89 ± 10.68 min vs. group M 7.62 ± 8.49 min, respectively; *p* = 0.74). Hemodynamic stability Group D patients had more cardiovascular stability in terms of (a) Intraoperatively, systolic (SBP) and diastolic (DBP) blood pressure were significantly lower in the Dex group than in group M. (b) HR was significantly lower in the Dex group compared to the morphine group at extubation.
Abu-Halaweh S et al., 2015, Jordan [24]	To determine whether the Dex infusion initiated immediately after laparoscopic bariatric surgery, offers an advantage over a morphine infusion to rescue morphine and paracetamol requirements over the first 24 postoperative hours.	Prospective, randomized, blinded study.	Group D: infusion of either 0.3 mcg/kg/h dexmedetomidine Group M: 3 mg/h Morphine Both groups had the treatment initiated immediately post-operatively, upon arrival to the ICU, and continued for the first 24 h period.	Sample size (N) = 60 Mean age (SD) = 33.5 years ± 9.5 Age (range) = 18–58 BMI (range) = 33.5–52.7 Mean BMI (SD) = 43.0 ± 4.5 Comorbid conditions = ASA I (86.7%) and II Pain scores (VAS) assessed upon ICU arrival were significantly higher in the Dex group than in the morphine group.	Gastric band (2%) Gastric bypass (35%) Sleeve gastrectomy (63%)	Analgesic requirement (paracetamol and morphine) -There were no significant differences in mean rescue paracetamol and morphine requirements. -In the first 24 postoperative hours, 19 patients in Group D and six patients in Group M required supplemental morphine. The mean supplemental morphine requirements in Group D were 6.1 ± 3.1 mg, whereas 4.7 ± 2.9 mg in Group M ((*p* = 0.34). -The total morphine administered was 6.1 ± 3.1 mg and 72.9 ± 2.2 mg for patients in Groups D and M, respectively (*p* < 0.0001). -There was no difference between groups with respect to the number who required paracetamol for moderate pain (60% Group D vs. 50% Group M, *p* = 0.44). -More group Dex (63%) than Morphine (20%) patients reported severe pain (VAS > 70) (*p* = 0.0007).	Postoperative pain intensity The drug-time interaction was not significant for pain scores (VAS), MAP, respiratory rate (RR), or arterial oxygen desaturation (SpO2) (*p* = 0.45, 0.71, 0.99, and 0.68, respectively). However, the analysis did reveal a significant effect for a time in all outcomes except RR (all *p*-values < 0.0001). There was a significant time-by-group interaction for HR over 24 h (*p* = 0.0038). Incidence and severity of PONV = Significantly more patients reported nausea or vomiting in the morphine group (*p* = 0.0063). Nausea was reported significantly more in the morphine group (63.3% vs. 26.7%, *p* = 0.0043); however, there was no difference between groups for vomiting (10.0% Dex group vs. 23.3% morphine group, *p* = 0.1659).	
Ziemann-Gimmel P et al., 2014, USA [25]	To assess whether opioid-free total intravenous anesthesia reduces postoperative nausea and vomiting in bariatric surgery.	Prospective, randomized parallel-group, single-blinded, single-center Study.	Classic group: patients underwent general anesthesia with volatile anesthetics and opioids. Intervention group: Total i.v anesthesia (TIVA) group, patients underwent opioid-free TIVA with propofol, ketamine, and dexmedetomidine. Loading dose of dexmedetomidine (0.5 mcg/kg i.v. and maintained with an i.v. Infusion of dexmedetomidine (0.1–0.3 mcg/kg/h)	Sample (N) = 119 Age: Classic (n = 59): 50.4 ± 12.4 TIVA (n = 60): 50.5 ± 13.7 BMI: Classic: 45.32 ± 6.97 TIVA: 44.15 ± 7.46	Gastric band (GB), laparoscopic Roux-en-Y gastric bypass (LRYGB), revision of an LRYGB, removal of GB then LRYGB (Conversion), and sleeve gastrectomy	Incidence and severity of PONV In the classic group, 37.3% reported PONV compared with 20.0% in the TIVA group [*p* = 0.04; risk 1.27 (1.01–1.61)] with a RR reduction of 46.4%. The absolute risk reduction was 17.3% (number-needed-to-treat = 6). The severity of nausea was different in both groups (*p* = 0.02), with the highest rate of retching in the classical group. In the Classic group, more patients experienced retching than in the TIVA group (Dex group). All the patients who reported retching rated the level of nausea as severe. Of the seven patients in the Classic group complaining of retching, five patients reported vomiting. There was no difference in the number of patients requiring antiemetic rescue medication (AERM) in the postoperative period or the number of AERM doses required. Postoperative pain intensity = The acceptable pain scores (*p* = 0.53) and pain scores on arrival on the ward (*p* = 0.66) were not different. Analgesic requirement The two groups’ average hydromorphone doses were equivalent in the postoperative period: 2.29 mg (±1.52 mg) and 2.08 mg (±1.17 mg), respectively (*p* = 0.40). Recovery time: There was no difference in the time from the end of the operation to PACU arrival time, 16 min (±13) vs. 15 min (±9) (*p* = 0.50), and no difference in the time it took patients to meet the discharge criteria from the PACU 44 min (±23) vs. 44 min (±19) (*p* = 0.92) in the Classic group vs. the TIVA group. Adverse effects: There were two adverse events in the TIVA group. One patient developed a second-degree AV block, and one patient developed hypotension in the PACU.		
Tufanogullari B et al., 2008, USA [18]	To test if Dex infusion would produce a dose- related reductions in the anesthetic and analgesic requirements in patients undergoing laparoscopic bariatric surgery. The secondary objectives were to determine if using Dex facilitated the recovery process and improved patient outcomes.	Prospective randomized, double-blind, placebo-controlled, dose-ranging study.	Patients were randomly assigned to one of four treatment groups: (1) The control group received a saline infusion during surgery. (2) The Dex 0.2 mcg/kg/h group (3) Dex 0.4 mcg/kg/h group (4) Dex 0.8 mcg/kg/h group	Sample size (N) = 80 Age range = 22–66 Control: 43 ± 16 Dex 0.2 47 ± 10 Dex 0.4 48 ± 9 Dex 0.8 40 ± 10 BMI = Morbid obesity Comorbid conditions = ASA II–III	Gastric banding, gastric bypass.	Extubation: Dex infusion, 0.2, 0.4, and 0.8 mcg, reduced the average end-tidal desflurane concentration by 19, 20, and 22%, respectively. However, it failed to facilitate a significantly faster emergence from anesthesia. Intra-operative hemodynamic: Values were similar in the four groups; arterial blood pressure values were significantly reduced in the Dex 0.2, 0.4, and 0.8 groups compared with the control group on admission to the PACU (*p* < 0.05). Recovery time: Did not differ among the four groups (controls, Dex 0.2, Dex 0.4, Dex 0.8); however, it was significantly reduced in the Dex groups (81 ± 31 to 87 ± 24 vs. 104 ± 33 min in the control group, *p* < 0.05). Analgesic requirements: The amount of rescue fentanyl administered in the PACU was significantly less in the Dex 0.2, 0.4, and 0.8 groups versus the control group (113 ± 85, 108 ± 67, and 120 ± 78 vs. 187 ± 99 mcg, respectively, *p* < 0.05). Incidence and severity of PONV: The overall incidences of postoperative emetic symptoms during the first 24 h after surgery were reduced in the Dex 0.2, 0.4, and 0.8 groups compared with the control group (25%, 30%, and 45% vs. 65%, respectively). Similarly, the need for rescue antiemetic drugs in the PACU was significantly reduced in all three Dex groups (30%, 30%, and 10% vs. 70% in the control group). Post-operative morphine consumption: PCA morphine requirements on PODs 1 and 2 were not different among the four groups. Postoperative pain intensity: Pain scores in the PACU and the average on PODs postoperative days 1, 2, and 7 did not differ significantly among the four groups. Hospital stay: The length of hospital stay did not differ significantly between the groups. Patient satisfaction did not differ significantly among the groups nor the quality of recovery scores and times to recovery of bowel function.		
Hassan S B et al., 2007, Egypt [26]	To evaluate the effect of dexmedetomidine on anesthetic requirements during surgery, hemodynamic, recovery profile, and morphine use in the postoperative period.	Prospective randomized, blinded study.	Group D (40 patients) received Dex (0.8 mcg/kg then 0.4 mcg/kg/h). Group P (40 patients) received normal saline (placebo) in the same volume and rate. Patients and investigators were blinded, but the anesthesiologist was aware of the condition of the treatment. The same surgeon performed all the surgeries.	Sample (N) = 80 Age(range) = 26–55 Group P: 29 ± 8 Group D: 30 ± 6 BMI = Morbid obesity Group P = 42 ± 5 Group D = 43± 6 Comorbid conditions = ASA II/III	Roux-en-Y gastric bypass (intraoperative), IV	Intraoperative hemodynamic stability: During anesthesia, MAP and HR significantly decreased in the Dex group compared with the placebo group. The total amount of intraoperative fentanyl required to maintain the hemodynamics was significantly lower in the Dex compared with the placebo group. The total amount of propofol required to maintain the target BIS (bispectral index) level was significantly lower in the dexmedetomidine group compared with the placebo group. Postoperative pain intensity = Pain scores at one hour and two hours, blood pressure, and HR were significantly lower in the Dex group in the PACU. Postoperative analgesic requirement The total amount of PCA morphine at two hours in the PACU and POD was significantly lower in the dexmedetomidine compared with the placebo group. Incidence and severity of PONV There was no difference in PONV incidence between groups. Recovery time = Duration to spontaneous respiration, adequate respiration, and safe extubation was significantly shorter in the Dex group compared with the placebo group.		

DEX: dexmedetomidine, DPB: diastolic blood pressure, HR: Heart rate, MAP: mean arterial pressure, MOASS: Modified observer’s agitation/sedation scale NRS: numerical rating scale, PACU: Post anesthesia care unit, PCA: Patient-controlled analgesia; POD: postoperative day, PONV: Postoperative nausea and vomiting, SBP: Intraoperatively systolic, TIVA: Total intravenous anesthesia.

## Data Availability

The datasets used and/or analyzed during the current study are available from the corresponding author upon reasonable request.
